# Examining the Relationship Between HIV-Related Stigma and the Health and Wellbeing of Children and Adolescents Living with HIV: A Systematic Review

**DOI:** 10.1007/s10461-023-04034-y

**Published:** 2023-03-14

**Authors:** Abbie Robinson, Aoife Cooney, Catherine Fassbender, David P McGovern

**Affiliations:** School of Psychology, Dublin City University, Dublin, Ireland

**Keywords:** Human immunodeficiency virus (HIV), stigma, wellbeing, children and adolescents

## Abstract

Human immunodeficiency virus (HIV) affects millions of people globally. The associated stigma remains a challenge for individuals living with HIV and children and adolescents face the additional challenge of withstanding the peer, pubertal and identity challenges associated with growing up. The current systematic review aimed to define and explore the major stigma-related challenges of children and adolescents from their own perspectives. A secondary aim was to identify any challenges distinct to childhood and adolescence. Studies included individuals aged 3 to 18 years who were aware of their status. Fifteen studies met inclusion criteria. Narrative synthesis was conducted on the included studies. Five analytic themes emerged describing major stigma-related challenges: disclosure-related anxiety, medication adherence, feelings of abnormality, mental health issues and social exclusion. Disclosure-related anxiety and feelings of abnormality appeared to be largely confined to the experience of children and adolescents. Many of the themes centred around peer influence, highlighting the need to belong in youth. Results suggest that youth require tailored interventions targeting their age-specific challenges.

**Supplementary Information:**

The online version contains supplementary material available at 10.1007/s10461-023-04034-y.

## Introduction

Human immunodeficiency virus (HIV) has been a major health issue worldwide since the disease was first recognized in 1981 [1]. Although the prevalence of HIV has been steadily declining in recent years due to advances in research and treatment, 38 million people were still reported to be living with HIV in 2019, with 1.8 million of those being children aged 0–14 years [2] and 1.7 million being adolescents aged 15–19 years [3]. The presence of HIV can have many adverse consequences on an individual’s health and wellbeing, with people living with HIV (PLHIV) individuals more likely to be diagnosed with depression, have a lower quality of life, lower physical and psychological wellbeing and lower wellbeing overall when compared with the general population [4, 5]. 

Stigma is another one of the major challenges associated with HIV. Stigma refers to the social process of labelling, stereotyping and prejudice causing separation, devaluation, and discrimination of specific people in a population [6]. In the early years of the HIV epidemic, the growing stigma towards PLHIV was highlighted as being an equivalent challenge to the disease itself [7]. Despite the prevalence of HIV, stigma is one of the major barriers in its prevention and treatment [8]. Lower levels of health and wellbeing associated with individuals living with HIV may be in part due to their experience of HIV-related stigma. Stigma can present itself in many forms for those living with HIV, particularly enacted stigma, anticipated stigma and internalised stigma. Enacted stigma refers to a negative public perception of those with HIV leading to discrimination and prejudice faced by the stigmatised population [9]. Anticipated stigma is the awareness one has of the negative social connotations towards HIV and the likelihood of experiencing prejudice or discrimination  [10]. Internalised stigma occurs when the individual living with HIV takes negative public perceptions and accepts them as self-truths, leading to feelings of shame, worthlessness and guilt [11]. Feelings of stigmatisation in people living with HIV have been associated with lower quality of life, mental health and wellbeing and reduced access to treatment [12]. The negative effects of HIV-related stigma on those living with HIV have been highlighted by a number of studies. For example, in a recent systematic review of HIV and stigma, stigma was shown to be associated with depression, reduced social support from friends, family and health services, lower medication adherence, lower usage of health and social services, anxiety, lower quality of life, worse physical health, increased emotional and mental distress and risky sexual behaviour [10]. These findings suggest that HIV-related stigma can negatively impact those living with HIV across a range of health-related outcomes.

One issue with the aforementioned review by Rueda and colleagues [10] is that the age range of the participants included in this analysis was not specified making it difficult to assess whether differences exist in the effects of HIV-related stigma observed in adults from those seen in children and adolescents. Childhood and adolescence represent periods of life that are inherently full of stressors and challenges, including building relationships, academic-related stress, self-esteem, puberty and physical and cognitive development [13] and these challenges could be exacerbated by the effects of HIV-related stigma. Furthermore, the desire to belong is distinctly present in youth, with pressure being put on younger individuals to fit in with others in society to avoid the effects of social turbulence which may persist into adulthood relationships [14]. Consequently, children and adolescents living with such a stigmatised disease as HIV may face more stressors in life, resulting in more negative consequences [15]. Such challenges that may uniquely affect children and adolescents living with HIV are sexuality shaming [16], caregiver HIV-related death [17], peer status disclosure [18] and elevated levels of bullying [19]. In line with anticipated stigma, medication adherence has been reported to be particularly low in adolescents, due to anticipation of stigma from friends and family if their status was disclosed [20], while similar results have been reported in younger adults when compared to their older counterparts [21]. This may lead to further physical health problems and the emergence of treatment-resistant strains of the virus [20]. Thus, a systematic review of the literature specific to children and adolescents is required to accurately gauge whether this negative trend extends to other stigma-related outcomes. Such a review could reveal important age-dependent differences in HIV-related stigma and its relationship on outcomes relevant to psychological wellbeing; should this be the case, this information could be used in the development of age-appropriate interventions targeting stigma. A recent systematic review highlighted a small number of studies in relation to interventions focused on self-stigma [22], so results from this review may help in the future development of these interventions.

The extent to which children and adolescents are subjected to and experience stigma is currently unclear, as there have been suggestions that the experience of HIV-related stigma may differ in childhood, depending on the age of the child affected by HIV [23]. Conflicting findings have been reported in this area and there is debate over the age at which the greatest HIV-related stigma is experienced [24, 25]. Deacon and Stephney [26] have suggested that this conflict may be the result of public attitudes towards the age at which a diagnosis is received. Some may view children living with HIV as a “blameless population” and “innocent”, based on the assumption that they contracted the disease perinatally and thus, are in receipt of empathy and kindness from the surrounding community. However, adolescents with HIV have also been viewed as “guilty” of their HIV transmission assumed to be a consequence of risky sexual behaviour [26]. Meanwhile, generational stigma and blame for their HIV serostatus has also been observed towards children living with HIV due to the perceived sexual promiscuity of their parents [26].

### Rationale

Many systematic reviews centred around HIV-related stigma collectively focus on all age ranges or on adults alone [10] and systematic reviews involving children tend to include children living without HIV and the stigma associated with their parents living with HIV [27]. To our knowledge, to date, no review has focused on the experiences of children and adolescents living with HIV (CALHIV). It is particularly important to address age and serostatus, as when an individual experiences a disease, it tends to become intertwined with one’s understanding of their identity and possibly mitigate the effects of stigma  [28]. For perinatally CALHIV, this concept is particularly important, as children are more likely to normalise stigmatising situations than their adult counterparts [29]. In order to gain a true insight into the stigma-related experiences of CALHIV, it is important that the voices of children and adolescents are central and actively involved in the research conducted in this area [30] in order to yield an accurate representation of their stigma-related experiences.

### Specific Objectives

This systematic review aims to combine and synthesise the evidence from studies of HIV-related stigma and its relationship to mental, physical and social wellbeing outcomes in children and adolescents living with HIV thereby providing a comprehensive understanding of the life of a child/adolescent living with HIV. The primary objective of this systematic review is the identification of the specific and major challenges faced by CALHIV to allow for insight to be gained regarding the societal and personal stigma they face. Targeted and tailored intervention programmes may also be directed towards negative outcomes they face due to these forms of stigma. A secondary objective of this review is to gauge if there is an age-dependency of HIV-related stigma within childhood and adolescence and to identify any differences between outcomes found in the present study for CALHIV and adults living with HIV. To this end, we compared the outcomes of CALHIV derived from the current review with those determined from a review that examined the impact of HIV-related stigma on wellbeing outcomes in adults [10].

## Methodology of Review

### Inclusion Criteria

To determine the inclusion criteria for studies to be included in the systematic review, the SPIDER tool was used [31]. The sample (S) domain required studies to include children and adolescents aged 3–18 years who have received a HIV diagnosis and were aware of their status. The phenomenon of interest (PI) is the relationship between levels of HIV-related stigma and any outcome measure associated with an aspect of physical, psychological or social health and wellbeing. This allows for the potential emergence of outcomes specific to children and adolescents, rather than just reflecting typical adult outcomes on a younger age group. The design (D) included methods that allowed for the reporting of the primary experiences of HIV-related stigma in those living with HIV, such as interviews, surveys, focus groups and questionnaires. The evaluation (E) outcomes will include the association between HIV-related stigma and experiences and feelings. This will be inclusive of, but not limited to, the main outcome measures of previous research; depression, social support from friends, family and health services, medication adherence, usage of health and social services, anxiety, quality of life, physical health, emotional/mental distress and sexual risk practices [10]. The research type (R) to be included are quantitative, qualitative and mixed-methods research presented in the form of published peer-reviewed journal articles. Furthermore, to be included in the review, there was a requirement for full-text articles to be available in English.

Considering the SPIDER process and the inclusion criteria, certain studies were excluded. Studies were excluded when full-text articles could not be obtained after exhaustive searches and contact with the authors. Studies were also excluded if critical data to the hypotheses of the study was missing from the full text. Studies that focused on the experiences of HIV-related stigma from a different perspective, for example a parent of a diagnosed child, were also excluded. Book chapters, editorials, letters, reviews, dissertations, opinion pieces, protocols and conference abstracts were all excluded.

See Table 1 for a summarised version of the inclusion/exclusion criteria in relation to SPIDER. Furthermore, “children/adolescents are aware of their HIV diagnosis” was added to the sample criteria to ensure the samples in studies were aware of their HIV diagnosis and thus aware of the stigma associated with it. No date range was selected in order to obtain a full overview of the available literature as, to the authors’ knowledge, no previous review has explored the links between stigma, wellbeing and HIV in childhood and adolescence.

**Table 1:** *SPIDER Table of Study Inclusion and Exclusion Criteria*

SPIDER	Inclusion criteria	Exclusion criteria
**Sample**	● Children/adolescents aged 3–18 years with a HIV diagnosis ● Children/adolescents are aware of their HIV diagnosis	● Studies including children/adolescents affected by HIV but do not have a diagnosis ● Those outside the age bracket of 3–18 years living with HIV ● Those unaware of their HIV diagnosis ● Studies which also include additional populations (e.g., caregivers) but do not distinguish between these groups in the results
**Phenomenon** **of Interest**	● Studies looking at HIV-related stigma and any outcome measure associated with an aspect of physical, psychological or social health and wellbeing.	● Studies that only analyse stigma or wellbeing outcome measures independently of one another ● Studies that look at the relationship between stigma and its associated behaviours through a non-wellbeing lens (e.g., stigma and the primary act of non-disclosure vs. stigma and the emotional impact of non-disclosure)
**Design**	● Questionnaire, survey, interview, focus group discussion (FGD)	● Individual case study
**Evaluation**	● Analysis of the relationship between HIV-related stigma and wellbeing outcomes experienced first-hand by a child/adolescent diagnosed with HIV	● Studies that look at the relationship between HIV-related stigma and wellbeing outcomes in children/adolescents from the point of view of a secondary party, such as a doctor or parent
**Research type**	● Quantitative: a measure of both stigma and of wellbeing is recorded by the CALHIV ● Qualitative: The effect of stigma on an aspect of wellbeing emerges as a main theme or if stigma emerges as a core focus within a main theme. ● Mixed-methods ● Peer-reviewed journal articles ● Full text available in English	● Book chapters, editorials, letters, reviews, dissertations, opinion pieces, protocols and conference abstracts

### Strategies to Minimise Bias

Multiple approaches were used to minimise bias. Two of the authors (AR and AC) conducted abstract screening, full-text screening and quality appraisal to reduce reporting bias. Furthermore, the PRISMA guidelines were used to guide the review, the PRISMA checklist and PRISMA flowchart were followed to ensure full transparency of the research [32].

Furthermore, the primary researcher engaged in reflexivity by drawing awareness to their cultural bias of sexual stigmatisation in Ireland [33] throughout the data extraction and analysis. CF and DPM also oversaw the finalisation of analytical themes, both of whom have a background in cognitive psychology and neuroscience, which should serve to further minimise bias in the analytical process.

### Search Strategy

Only peer reviewed, published literature available through databases were considered for the review. Databases searched were Embase, PsycInfo, PubMed and CINAHL Complete. Embase, PubMed and CINAHL were chosen to reflect the health-related aspect (i.e., HIV) of the review. PsycInfo was chosen to capture the behavioural and psychological aspects of stigma. All searches were conducted on January 21st, 2021. Given that HIV and stigma are wide areas of interest alone, a search strategy was implemented.

The subject of the search terms was largely devised based on the “S” and “PI” aspects of SPIDER to prevent narrowing down the search too much. “D”, “E” and “R” were considered in further detail during the title and abstract screening stage. The search terms were decided upon using keywords from relevant studies [e.g., 17, 24, 34] and following consultation with the subject librarian for psychology from the Dublin City University library. The search strategy was to combine two searches, one related to children and adolescents living with HIV and one related to HIV-related stigma. The aim was to identify studies with both of these components and thus identify possible outcome measures from there. In each independent search, search terms were combined using the Boolean operator “OR” in order to broaden the search. Both independent searches were then joined using the Boolean operator “AND” in order to combine the two independent searches and narrow the overall search to identify references containing all of the words entered. The wildcard of * was used within the appropriate databases to include all potential variants of terms with different spellings or suffixes. An example of the PsycInfo search strategy can be seen in Appendix A.

The possibility of inclusion of hand searched studies was considered. Hand searching involved the examination of bibliographies from key papers as well as using the “related articles” function in Google Scholar. No such studies emerged from these searches.

### Details of Methods

The review team consisted of two reviewers (AR and AC) and two supervisors (DPM and CF). References were downloaded onto reference manager Zotero [35] and duplicates were excluded. The remaining references were imported into Covidence [36] for screening. Dual screening was conducted to ensure best practice in the reduction of error rates and chance of bias. The two reviewers screened the titles and abstracts to identify any potentially relevant studies from the searches. Studies identified as being potentially relevant to the review were moved onto the full text screening stage in which the full texts were screened by the two reviewers against the inclusion/exclusion criteria. The authors of any studies identified without an openly available full text were subsequently contacted for a copy. In the case that there was a disagreement among the two reviewers in terms of the inclusion of a study, the reviewers met and resolved the disagreement through discussion and consensus. At the outset of the review, it was agreed that where a consensus could not be reached between the two reviewers, the supervisor would make the final decision regarding the inclusion/exclusion of a study; however, this step was not required in the current review.

### Data Extraction

Information extracted from each study was placed in a data extraction table in Microsoft Excel. Data extracted from eligible articles included the (1) author, (2) year, (3) country, (4) study design, (5) method of data collection, (6) aim/objective, (7) participant recruitment, (8) sample size, (9) participant descriptors, (10) other groups involved, (11) type of stigma addressed, (12) outcome measure, (13) any scales used, (14) the key findings, statistics and key quotes and (15) conclusion. If any data was missing from a journal article, the study authors were contacted for further information and clarification. In cases where data could not be obtained due to non-recording or non-response, the relevant field was marked as “not stated”.

### Quality Assessment

After extraction, quality appraisal was conducted by the two reviewers using an Excel spreadsheet to evaluate the risk of bias of included studies. As quantitative, qualitative and mixed-methods studies were included in the review, two quality appraisal tools were selected for use.

The quantitative quality appraisal tool is adapted from Dunne and colleagues [37] and Jefferies and colleagues [38] and consists of a series of questions designed to assess the quality of quantitative studies. Criteria for quality were assigned an answer of ‘yes’, ‘partial’, ‘no’ or ‘don’t know’, which mapped onto scores of 2, 1, 0 and 0, respectively, with higher overall scores indicating a higher quality study. Examples of the criteria are “clearly stated aims” and “statistical methods described”.

The qualitative quality appraisal tool used was the CASP Qualitative Checklist [39]. Scoring followed a ‘yes’, ‘no’, ‘can’t tell’ approach. An example of questions asked are “is a qualitative methodology appropriate?” and “was the data collected in a way that addressed the research issue?”. As there is no established scoring system for this tool, question 8 “was the data analysis sufficiently rigorous?” was selected to be used as a proxy for quality [see 40].

### Data Synthesis

Data synthesis was emergent and was decided upon following the final full-text studies being selected. This was due to the limited literature available on this topic and the exploratory nature of this systematic review, as it was primarily designed to provide an insight and overall description of the health and wellbeing of CALHIV and their relationship with stigma.

Narrative synthesis was used to synthesise information from the number of different types of study designs included. The method for thematic synthesis was used to approach this [41]. This consisted of three steps: (1) Line-by-line coding of the extracted data, (2) The formation of descriptive codes and (3) The formation of analytic themes. This approach was selected to allow the emergence of higher order themes that go beyond what is addressed in the original studies. This was carried out by hand. A sample of the line-by-line coding that aided theme formation can be seen in Appendix B. This process was conducted in an inductive manner, allowing for the themes to emerge from the text. No themes were identified before coding commenced, due to previous systematic reviews being adult-focused, as it was hypothesised that themes for CALHIV may differ from previously identified themes.

## Results

**Fig. 1:**
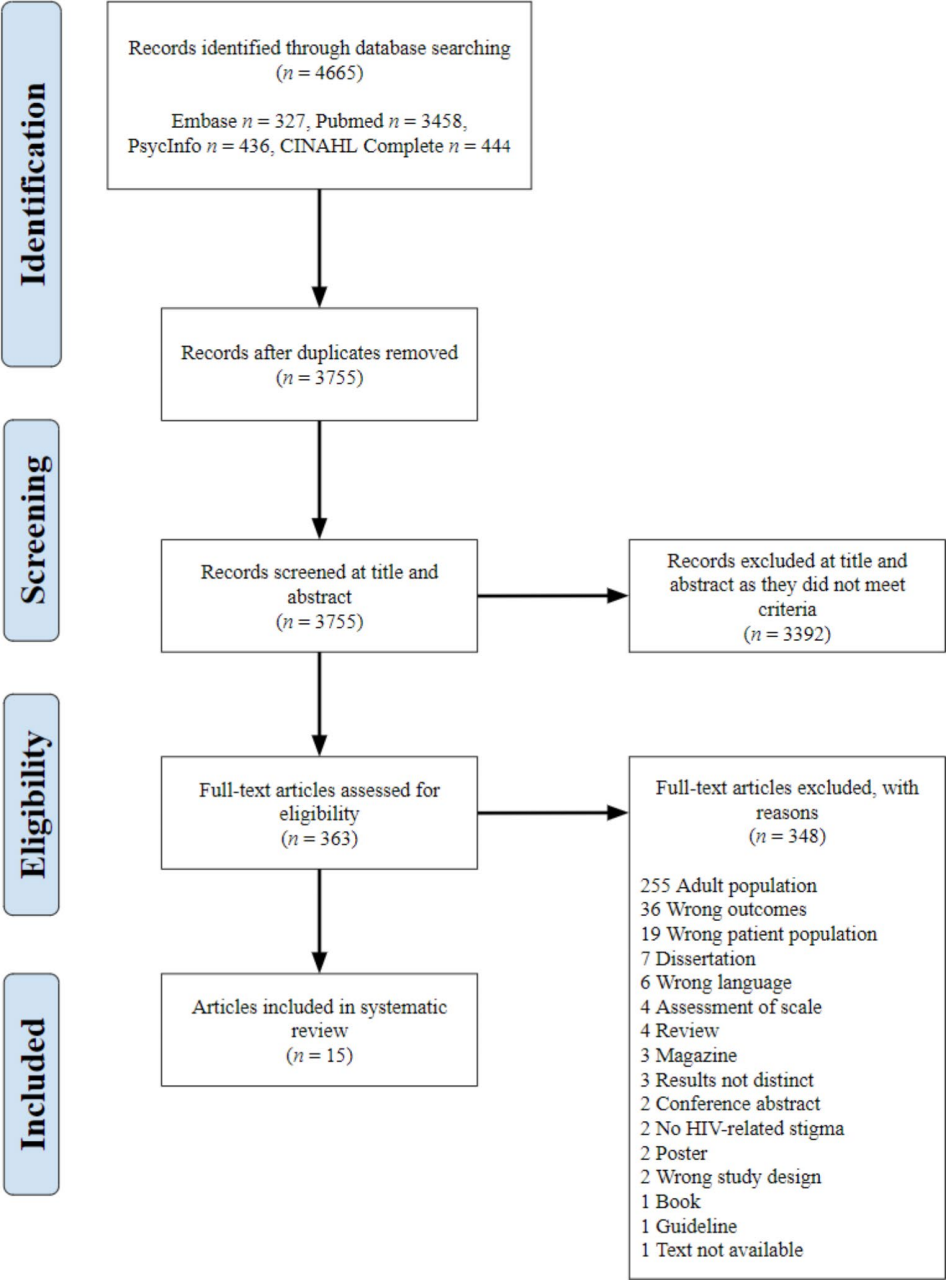
*PRISMA Flowchart*

The four databases searched identified 4665 records for potential inclusion, which resulted in 15 articles included in the review following the removal of duplicates and abstract and full-text screening (see Fig. 1). Duplicates accounted for 910, while abstract screening identified that 3392 articles did not meet the inclusion criteria. During abstract screening 9.6% (360) of assessments resulted in conflicts between the two reviewers, primarily due to the age of samples included in the study, with all conflicts being resolved through discussion between the reviewers. A further 348 articles were excluded at the full-text screening stage as they did not meet the inclusion criteria. One article [42] was excluded due to the unavailability of the text after contacting the author. During this screening, 8.5% (21) of the texts resulted in conflicts between the reviewers at this stage, most of which related to study outcomes and patient populations. All were resolved through discussion between the reviewers.

### Study Characteristics

Across the 15 studies included there were 1275 children and adolescents aged between 7 and 18 years. The broadest age range studied was 8–18 years [43]; the narrowest was 9–11 years [44] and 11–13 years [45]. All studies included CALHIV who were aware of their status. Of the 15 studies included, eight studies only included perinatally-acquired participants [43, 44, 46–51], three studies did not record this information but deemed it probable that their sample had HIV from birth [52–54], while the remaining four studies did not provide specific detail on this matter [45, 55–57]. Included articles were published between 2005 and 2019. Qualitative (*n* = 10) and quantitative studies (*n =* 5) were included. Studies were conducted in Uganda (*n* = 3), Kenya (*n* = 2), Malawi (*n* = 2), South Africa (*n* = 2), Brazil (*n* = 1), Canada (*n* = 1), Namibia (*n* = 1), Sweden (*n* = 1), Thailand (*n* = 1), and the United States (*n* = 1). Participants were primarily purposefully recruited from HIV-specialised clinics (*n* = 14) and in one case participants were recruited from a HIV group home [53]. All quantitative studies used questionnaires for data collection and one study also employed an interviewed scale [52]. Qualitative data collection primarily relied on interviews and focus group discussions (FGD). Five of the studies generally explored children and adolescents’ experiences; five focused on psychosocial factors; three focused on medication adherence; one focused on disclosure; and one focused on health-related quality of life (see Tables 2 and 3). The characteristics of the included studies are further described in Tables 2 and 3 and Appendix C.

**Table 2:** *Characteristics of Qualitative Studies*

Author (Year), Country	Study Design	Participants	Age Range	Focus	Type of Stigma addressed	Stigma-related Findings	Quality Assessment Rating
Roberts (2005), USA [57]	Interviews	9	7–12	Medication adherence	Anticipated & internalised (inferred)	Stigma emerged as its own theme affecting medication adherence. Reminder of HIV/AIDS also emerged as a theme, highlighting the alienation children felt due to having a stigmatised disease.	Higher
Friedman-Nestadt et al. (2018), Thailand [48]	FGD & Interviews	10	13–16	Psychosocial factors	Anticipated & internalised (inferred)	Two themes addressed stigma. Adherence issues and disclosure both encompassed fear and secrecy.	Lower
Madiba & Mokgatle (2016), South Africa [51]	Interviews	37	12–18	Disclosure	Anticipated & enacted (inferred)	Two themes addressed stigma. Keeping the secret addressed hiding the status. Experience of isolation resulted from breaking this secrecy. Self-disclosure addressed fears of rejection and isolation from peers and romantic partners.	Higher
Lima & Pedro (2008), Brazil [50]	Interviews	4	10–14	General experiences	Internalised (inferred)	Non-acceptance of such a stigmatised disease. Negative emotions and feelings of inequality. Even when moved into the acceptance phase, anguish was still experienced.	Lower
Phuma-Ngaiyaye & Dartey (2015), Malawi [56]	Interviews	10	10–14	General experiences	Anticipated & enacted (inferred)	Fear of stigmatising by their peers, leading to non-disclosure. Enacted stigma in the family environment.	Higher
Kawuma et al. (2014), Uganda [45]	Interviews	26	11–13	Medication adherence	Anticipated & internalised (inferred)	Fear of being seen by others was identified as a theme, encouraging secrecy. This led to missed doses.	Higher
Abubakar et al. (2016), Kenya [46]	Interviews	12	12–17	Psychosocial factors	Anticipated (inferred)	Poor handling of disclosure discussed how partial disclosure appeared to be the preferred method of disclosure, leading to unfavourable outcomes. Many feared full disclosure would cause rejection and isolation, resulting in medication adherence issues.	Higher
Fournier et al. (2014), Uganda [53]	Photovoice & FGD	13	12–18	General experiences	Enacted & internalised (inferred)	Stigma and discrimination showed children in the home were signalled out as having HIV. Suicidality was alluded to as a result. Emotional challenges identified anger, bullying, abuse, accusation of criminal activity.	Lower
McHenry et al. (2017), Kenya [55]	FGD	40	10–15	General experiences	Anticipated, enacted & internalised (stated)	Anticipated stigma most frequently reported, mostly regarding relationship loss. This affected medication adherence and subsequent physical health. Participants reported low self-esteem, being unworthy and hating themselves. Fewer participants reported experiencing stigma.	Higher
Fielden et al. (2006), Canada [47]	FGD & Interviews	10	9–16	General experiences	Anticipated, enacted (stated) & internalised (inferred)	Social stigma was frequently associated with fear, secrecy, trust, disclosure and isolation. Mental health highlighted the atypical worries possessed by youth living with HIV, such as partner rejection.	Higher

**Table 3:** *Characteristics of Quantitative Studies*

Author (Year), Country	Study Design	Participants	Age Range	Focus	Type of Stigma addressed	Stigma-related Findings	Quality Assessment Rating
Rydström et al. (2016), Sweden [43]	Questionnaire	58	8–18	Health Related Quality of Life	Anticipated & internalised (scale)	Strong negative association found between stigma and health relation quality of life.	Good
Gentz et al. (2017), Namibia [54]	Questionnaire	99	12–18	Psychosocial factors	Anticipated, enacted & internalised (scale)	Correlational findings showed a positive relationship between total stigma and total difficulties, emotional symptoms, peer problems, and conduct problems. Multiple regression found that stigma was a significant predictor for both total mental health difficulties and emotional symptoms.	Good
Kim et al. (2017), Malawi [49]	Questionnaire	519	12–18	Medication adherence	Anticipated stigma (scale)	9.8% of individuals who reported non-adherence reported stigma outside the home as a barrier. 5.2% of them reported stigma inside the home as a barrier.	Acceptable
Ashaba et al., (2018), Uganda [52]	Questionnaire & interviewed scales	224	13–17	Psychosocial factors	Internalised (scale)	Bivariate analysis showed major depressive disorder and suicidality associated with internalised stigma. In a multiple regression suicidality had a significant association with internalised stigma.	Good
Hoare et al. (2019), South Africa [44]	Questionnaire	204	9–11	Psychosocial factors	General stigma (scale)	HIV-related stigma was found to be most associated with depression, anger and disruptive behaviour.	Good

### Quality Appraisal

The ten qualitative studies were assessed using the CASP Checklist [39]. These results can be seen in Table 4. Question 8 of the CASP - “how rigorous was the data analysis”- was selected to determine quality [40]. Seven studies were deemed of higher quality and three of lower quality. Lower quality studies did not consider the researcher’s role [48], did not have a second reviewer [48, 50], or did not have enough funding to allow for thorough analysis of the data [53]. The five quantitative studies were assessed using Dunne and colleagues [37] criteria. The results of this assessment can be seen in Table 5. Four of the five quantitative studies were deemed to be good quality and one was deemed to be acceptable quality. Kim and colleagues [49] was deemed of acceptable quality due to partial discussion of the results and recruitment strategy. However, no higher weight was given to studies of a higher quality, given the subjectivity of qualitative research and the similar outcomes found across both the quantitative and qualitative studies.

**Table 4:** *CASP Checklist*

Criteria	Roberts, 2005 [57]	Friedman-Nestadt et al., 2018 [48]	Madiba & Mokgatle, 2016 [51]	Lima & Pedro, 2008 [50]	Phuma- Ngaiyaye et al., 2015 [56]	Kawuma et al., 2014 [45]	Abubakar et al., 2016 [46]	Fournier et al., 2014 [53]	McHenry et al., 2017 [55]	Fielden et al., 2006 [47]
Was there a clear statement of the aims of the research?	Y	Y	Y	Y	Y	Y	Y	Y	Y	Y
Is the qualitative methodology appropriate?	Y	Y	Y	Y	Y	Y	Y	Y	Y	Y
Was the research design appropriate to address the aims of the research?	Y	N	Y	Y	Y	Y	Y	Y	Y	Y
Was the recruitment strategy appropriate to the aims of the research?	Y	Y	Y	N	Y	Y	Y	Y	N	Y
Was the data collected in a way that addressed the research issue?	Y	Y	Y	Y	Y	Y	Y	Y	Y	Y
Has the relationship between the researcher and participants been adequately considered?	N	N	N	N	N	N	N	N	N	N
Have ethical issues been taken into consideration?	N	N	Y	Y	Y	N	Y	Y	Y	Y
Was the data analysis sufficiently rigorous?	Y	CT	Y	CT	Y	Y	Y	CT	Y	Y
Is there a clear statement of findings?	Y	Y	Y	Y	Y	Y	Y	Y	Y	Y
How valuable is the research?	CT	CT	Y	N	CT	Y	Y	CT	CT	N

Score: Y = Yes, N = No, CT = Can’t Tell

**Table 5:** *Dunne and Colleagues Criteria*

Criteria	Kim et al., 2017 [49]	Rydström et al., 2016 [43]	Ashaba et al., 2018 [52]	Gentz et al., 2017 [54]	Hoare et al., 2019 [44]
Clearly stated aims	Y	Y	Y	P	Y
Participant eligibility and recruitment strategy clearly documented	P	Y	P	Y	Y
Main features of population/design described	Y	Y	Y	Y	Y
Non-responders (and non-participants) described	N	Y	N	N	N
Presence of a control group	N	N	N	N	Y
Main limitations identified and acceptable	Y	Y	Y	Y	Y
Sample size justified	N	N	Y	Y	Y
No evidence of selective reporting of results	Y	Y	Y	Y	Y
Statistical methods described	Y	Y	Y	Y	Y
Statistical methods appropriate	Y	Y	Y	Y	Y
Measures relevant, validated and described adequately	Y	P	Y	Y	P
Results discussed adequately	P	Y	Y	Y	Y
Total	16	19	19	19	21

Score: Y = Yes (Score of 2), P = Partial (Score of 1), N = No (Score of 0)

Scoring: 17–24 = good quality; 9–16 = acceptable quality; 0–8 = low quality

### Thematic Synthesis

Following thematic synthesis [41] of the included studies, five major challenges associated with HIV-related stigma were identified. These were disclosure-related anxiety, medication adherence, feelings of abnormality, mental health issues and social exclusion.

#### *Disclosure-related Anxiety*

A theme to feature across multiple studies was the association between stigma and disclosure-related anxiety, appearing in eight studies. Disclosure-related anxiety refers to the experience of youths’ fear and worry associated with the thoughts of revealing their HIV status to others. This emerged as a separate theme to mental health issues due to its consistency in the research and the explicit association illustrated between fear and disclosure. Anticipated stigma was typically stated or inferred from this challenge, rather than the internalised stigma typically associated with mental health issues. Rydström and colleagues  [43] found that anticipated stigma associated with disclosure concerns was more profound than negative self-image and public attitudes. Fear was a prominent code identified in all instances of disclosure-related anxiety. Children and adolescents reported fears of stigma, rejection and isolation from their peers if they were to disclose in five of the included studies [47, 48, 51, 55, 56]. Three studies involving older participants noted how this fear also impacted romantic relationships, with constant worry regarding partner rejection upon finding out their status ((9-16years), [47]; (13-16years), [48]; (12-18years), [51]). Some studies viewed secrecy as a form of protection from such negative consequences of disclosure [46, 56]. In two studies, the practice of such secrecy was advised by immediate family in some cases [45, 51]. However, while trying to avoid negative consequences through secrecy, this acts as a source of constant worry and suffering for children and adolescents due to the evoked disclosure-related anxiety. This state of worry is reflected through a quote from a 13-year-old male participant *“I do not tell people…they can start discriminating me. They can tell their friends, others do not keep secrets.”*  (p360) [56]. Some participants reported engaging in partial disclosure, rather than complete secrecy [46, 48]. However, this too resulted in complicated relationships and further anxiety [46].

#### *Medication Adherence*

Medication adherence acts as an umbrella term to cover all stigma and medication-related challenges and was identified in seven studies. This emerged to be more complex than the simple act of missing a dose. Medication was viewed by children and adolescents as a signifier of their HIV status [45, 48, 57]. This led many children and adolescents to take their medication only when in private locations, away from the eyes of their peers [45, 46, 55, 57] or resorting to cover-up stories regarding the true purpose of their medication [57]. These fear-motivated behaviours were directly or inferentially linked to anticipated stigma and a desire to hide their status from others: *“Children [from the neighbourhood] would come [to my] home so early in the morning and when the time for taking drugs comes I would take it while they are there and when they see me taking it they ask me and when I tell them they start teasing me. So when they come I don’t find time to take it yet even the tins [with the drugs] are in the sitting room”* (11-year-old male; p191) [45]. In a study by Kim and colleagues [49], fear of stigma outside the home was reported as a barrier to medication adherence, albeit this was less frequently reported by participants. However, forgetting was a significant barrier to adherence, with 90% of participants citing lapses of memory as a reason for their failure to take medication. Kawuma and colleagues [45] found that missed doses due to forgetting related to the anticipated stigma of medication as a HIV signifier, which in turn led to the child or adolescent not receiving reminders to take their medication when in the company of their peers. The psychological stress the individual is placed under due to stigma was also noted to cause physical effects due to missed doses, including weight loss and ill appearance [55].

#### *Feelings of Abnormality*

While not an overarching theme typically reported in individual studies, the thematic synthesis revealed intense feelings of alienation due to living with such a stigmatised disease. Feelings of abnormality were identified in six studies. This appeared to stem from internalisation of stigma among children and adolescents. Non-acceptance of their diagnosis and anguish related to its lifelong effects and care was a major challenge identified [45, 50]. Children and adolescents expressed a desire to be normal and like their peers [47, 51]. However due to their stigmatised label, feelings of being unequal to their peers prompted emotional turmoil while many individuals also expressed feeling unworthy [50, 55]. Furthermore, the additional challenge of medication adherence acted as a constant reminder to some participants that they were not like other children [45, 57]. This appeared to impede any moments of normality for the CALHIV, as illustrated by a 12-year-old female participant, “*sometimes, I’ll wake up thinking I’m a normal kid, and my Grandma saying, ‘come take your medicine.’ And then I’ll think, ‘Oh, man, again.’ And so, I don’t feel so good.”* (p240) [57]. CALHIV are trying to live a “normal life”, yet this appears to be constantly inhibited by the reality of living with a life-long disease. Even individuals who had reached the acceptance phase of their diagnosis still expressed anguish towards their routines, suggesting a constant battle with alienation [50]. Children and adolescents appeared to relish the rare moments where they did not have to acknowledge that they were different to their peers, such as at camp for HIV-affected children, where their status was not met with stigma [47]. This further highlights the desire and importance of normalcy in the life of a child or adolescent living with HIV.

#### *Mental Health Issues*

Mental health issues emerged from eight studies. Many varying levels of mental anguish were observed, from general sadness to suicidality. These were grouped together to collectively form the theme of mental health issues. Most commonly reported in qualitative studies were painful feelings of worthlessness, sadness, discomfort, stress, hopelessness, low self-esteem and depression [43, 50, 53, 55, 56]. McHenry and colleagues (p6) [55] found that “participants often described feelings of ‘hating themselves’ and ‘insulting themselves in their hearts’”. Three of the five quantitative studies focused on these psychological aspects of stigma and found similar results. Stigma was found to be significantly related to depression, anger and disruptive behaviour [44]. Internalised stigma in particular was found to be a strong predictor of depression [52], total mental health difficulties, emotional symptoms and conduct problems [54]. Children appeared to apply the public perception of HIV to themselves, resulting in emotional turmoil. Elements of negative emotion were felt towards parents of the children and adolescents, ranging from grief [53] to betrayal [50]. Some children faced discrimination for losing a parent to HIV/AIDS, while others wondered why their parent would burden them with such a disease. In some instances, the internalised stigma was so intense it evoked feelings of suicidality [53], with a significant positive association between internalised stigma and suicidality identified in Ashaba and colleagues [52] study. There was a large degree of heterogeneity in the reporting of mental health issues across studies, but the overall commonality was the negative relationship between stigma and psychological wellbeing.

#### *Social Exclusion*

While the prior themes centred around internalised and anticipated stigma, children and adolescents also reported experience of enacted stigma, albeit to a lesser degree. Elements of social exclusion were found in seven of the fifteen studies. Social exclusion appeared to occur in family, peer and community environments and involved the singling out of the child or adolescent living with HIV. Teasing, bullying and isolation from peers was a common issue reported after disclosure [43, 45, 50, 53, 54]. For some, this translated over to the home environment, with reports of differential treatment and neglect from family members [50, 53, 56]. Some children experienced being stripped of their basic needs, due to abandonment and neglect, such as abandonment by rich relatives [53] and denial of food or drink due to fear of contamination, *“sometimes my sister in-law was not giving me food…she was also telling me not to use her water, maybe because I have HIV. Then I just ran away from home and went to my uncle.”* (14-year-old girl; p361) [56]. Some children and adolescents also experienced mocking by family members due to their appearance and medication [56]. Community actions in which the children and adolescents were pointed out in crowds, misrepresented in the media, accused of crimes or had their death speculated upon also appeared to cause much emotional turmoil [48, 53, 56]. Furthermore, in some cases, discrimination from the family led to exclusion from peers, “*even when we are playing with friends, she [sister] tells them not to play with me, because I am thin.”* (13-year-old female; p361)  [56]. While enacted stigma was not an occurrence in the majority of children and adolescents’ lives, for those experiencing it, enacted stigma served as a major challenge with negative psychological and social consequences.

## Discussion

The primary objective of this review was to explore and define the major challenges associated with HIV-related stigma from the perspectives of children and adolescents living with HIV. Through the identification, inclusion and thematic synthesis of 15 studies, five themes emerged as major challenges: disclosure-related anxiety, medication adherence, feelings of abnormality, mental health issues, and social exclusion. These themes indicate many additional unfavourable outcomes children and adolescents may have to face following the diagnosis of a stigmatising disease, further to and perhaps intertwined with the many stressors already present in child and adolescent development.

The emergence of mental health issues as a theme appeared to be rooted in internalised stigma. This further adds to the literature surrounding the many negative impacts that stigma has consistently demonstrated on mental health [e.g., 10–12]. Many children and adolescents reported milder symptoms, such as low mood. However, the impact of stigma led some children and adolescents to experience suicidal thoughts. Previous reviews noted a sparsity of interventions targeting stigma, in particular internalised stigma [22, 58]. Such vast and varying mental anguish may be the result of poor attention towards internalised stigma interventions, possibly allowing mild anguish to grow into more sinister suicidal thoughts.

Enacted stigma, such as bullying and teasing, was found to occur across family, peers and the community, resulting in social exclusion of the individual with HIV. The findings regarding social exclusion opposes the idea that children and adolescents are seen as an innocent population, deserving of empathy, as suggested by previous research [24, 25]. Despite the majority of studies involving perinatally-acquired individuals, bullying, neglect and stereotyping was reported in almost half of the studies. The targeted bullying regarding the death of their parents also reflects a society in which generational blame accompanies HIV diagnoses [26]. Despite the emergence of social exclusion as a theme, enacted stigma was less represented by the data. This may largely be attributed to the lack of disclosure of status from children and adolescents, thus reducing their risk of experiencing enacted stigma.

The period of childhood and adolescence is filled with a great need to fit in among peers [14, 59]. The emergence of the theme ‘feelings of abnormality’ emphasises the importance of this in the lives of CALHIV. The desire to feel ‘normal’ is a classic characteristic of puberty and the ‘abnormality’ of having a stigmatising disease appears to drastically affect the wellbeing of these young people. For example, they are not only faced with the classic stressor of building relationships [13], but they are also burdened with the constant fear of rejection upon disclosure to a friend or partner. Thus, disclosure-related anxiety may be linked to feelings of abnormality in children and adolescents, as CALHIV appear to experience relentless anxiety regarding their secret being revealed. Previously, parents have reported delaying status disclosure to their CALHIV, due to fear that they will not keep it a secret [60]. However, the current findings directly contrast this, with children actively hiding their status from peers. This further highlights the need for children and adolescents’ perspectives to be incorporated into HIV research.

The link between anticipated stigma and issues with medication adherence in this review support previous empirical findings highlighting the role of stigma and social barriers in medication nonadherence in adolescents and young adults [20] and further supports the theory that medication adherence is generally poorer among younger populations with HIV [21]. It should be noted that some researchers have suggested that demographic characteristics do not act as reliable predictors of adherence [61]. However, the current findings suggest that the need for belongingness that shapes childhood and adolescence plays a role in the complex nature of medication adherence. By actively delaying doses around peers, individuals with HIV may be at more risk of forgetting dosages, rather than just delaying their intake until alone. This highlights the importance of trusted adults in children’s lives to help manage their medication. Furthermore, the importance of centring research around children and adolescents’ experiences is highlighted here, as parents were generally unaware that their children missed doses [57].

Given that the majority of the studies focused on perinatally-acquired participants, it could be hypothesised that those living with HIV since birth would suffer less from stigma-induced outcomes, due to normalisation of the stigmatising elements associated with an early HIV diagnosis [28, 29]. However, no such distinction was identified between those who acquired HIV perinatally and those participants for which this status was unspecified. This calls into question the extent to which children and adolescents normalise stigmatising situations, given that stigma prevailed as an issue for all participants. This discrepancy between the previous theoretical findings and the current findings could be due to a number of factors, including enforced secrecy from an early age [45, 51] or the delayed disclosure of status to the child living with HIV [62]. Rather than their status being normalised from a young age, both situations may create an air of stigmatisation, secrecy or abnormality in the individual’s immediate environment. From the current results, it is clear that stigma plays a significant negative role in CALHIV’s lives, irrespective of when they contract the virus.

### Child-Specific Challenges of HIV

A secondary objective of the review was to identify if there is an age-dependency of HIV-related stigma such that outcomes might differ between children and adults with HIV. To date, the only systematic review addressing HIV-stigma and wellbeing in individuals with HIV was conducted without a specified age range [10]. There were a number of parallels between Rueda and colleagues [10] systematic review and the current review. Strong ties between stigma, depression and medication adherence were found for both children and adults, suggesting that depression and issues relating to medication adherence occur regardless of age; however, the current findings also suggest nonadherence may be a bigger issue for younger age groups. This area may be worthy of further research to establish whether this difference in medication adherence represents a substantive issue for younger individuals living with HIV.

Stigma was found to have only a weak relationship with general mental health-related anxiety in the review by Rueda and colleagues [10]. Disclosure-related anxiety was not explored within this review’s outcomes. However, disclosure-related anxiety was a prominent challenge identified by children and adolescents living with HIV across many of the included studies in the current review [e.g., 47, 51, 56]. This indicates that disclosure-related anxiety may act as a major challenge experienced by CALHIV that is distinct from the adult experience. A systematic review that developed a model of disclosure-related anxiety highlighted HIV-related stigma in the development of core beliefs as a key determinant of disclosure-related anxiety [63]. Core beliefs fostered at a young age have been found to have an enduring influence on the self [64]. When considering this through the lens of the current systematic review, it could be argued that the instilling of secrecy from a young age by parental figures may increase the likelihood of the development of intense core beliefs and place children and adolescents at significantly higher risk of disclosure-related anxiety. Furthermore, Rueda and colleagues [10] identified a weak link between stigma and sexual risk behaviours. While sexual risk behaviours were not identified in the current cohort, it was identified in one study that non-disclosure to partners may occur due to disclosure-related anxiety [48]. This is a serious risk as adolescents grow older and become sexually active, and as such, warrants further study to establish whether non-disclosure is indeed likely to occur in this cohort. In particular, the link between non-disclosure and medication adherence must be investigated, as missing doses in an attempt to hide status in sexually active populations may increase transmission of HIV.

Usage of health services was a major stigma-related challenge found across ages [10]; however, no such challenge relating to health service usage was found in the current review. This may be due to parental figures being responsible for scheduling healthcare services rather than the participants themselves. If this is the case, stigma relating to healthcare usage may become more of an issue when transitioning from child to adult services. Such studies exploring this challenge were excluded from the current review due to age criteria; however, this issue may merit a future review in its own right.

Another key difference between the current findings and those of Rueda and colleagues [10] was that feelings of abnormality were also confined to children and adolescents. This may be due to the high importance adolescents place on fitting in with their peers, particularly in school settings [14, 59]. While there definitely appears to be similarities across ages, disclosure-related anxiety and feelings of abnormality appear to be particularly prominent in children and adolescents living with HIV. However, due to the heterogeneity across studies and reviews, future research is recommended to further explore these distinctions.

### Strengths and Limitations

A major strength of the current review was its focus on HIV-related stigma from the perspectives of CALHIV. It is vital that the voices of young people are listened to in the research surrounding them in order to gauge a true understanding of their first-hand experiences [30], rather than attempting to infer what children are feeling on the basis of portrayals from parents and/or caregivers. In this respect, the current review focused on an area of research in its infancy and where the literature is, at present, limited in nature. However, one potential consequence of the exclusion of studies that reported amalgamated results from individuals with HIV and third parties (e.g., carers) is that crucial insights may have been overlooked, due to the perspectives of children/adolescents being indistinguishable from other sample groups and unable to be attributed solely to the child/adolescent’s experience [65–67]. This is an important consideration that researchers should make in the reporting of future research. Furthermore, two of the included studies noted that children and adolescents found it difficult to speak about certain situations, such as school experiences [50, 53]. This makes it difficult to establish if a full understanding of the child/adolescent’s experience with stigma was recorded. However, from the findings it is evident that placing a spotlight on children and adolescents allowed for key themes to emerge. Thus, this area must focus on the continued comfort and support for children and adolescents involved in the research to maximise the accuracy and value of the data collected.

However, while narrative synthesis was the most appropriate method to employ in the present review, due to the heterogeneity found amongst studies, this increased the risk of subjectivity and reporting bias due to data extraction and synthesis only being completed by one reviewer [68]. However, this risk of bias was mitigated through the review supervisor (DPM) overseeing the review and the dual control conducted in earlier parts of the review.

The purposeful sampling technique employed by the majority of included studies, limits the generalisability of the results and may have left recruitment in the individual studies open to researcher bias. However, CALHIV remain a very niche population and thus, purposeful sampling was warranted in such situations. Furthermore, the majority of the included studies were qualitative, meaning their sole purpose was not to generalise. As such, this review may help guide and inform future studies and interventions targeted towards these specific challenges rather than generalising to the entire population living with HIV per se. As the majority of the participants had perinatally-acquired HIV, findings may be more applicable to these individuals. More specific challenges may be applicable to children and adolescents who acquired HIV sexually, particularly due to the high likelihood of sexual assault and its own set of challenges [69]. Furthermore, ten of the fifteen studies included in the review were conducted in an African population. While this may appear to limit generalisability further, the majority of child and adolescent HIV cases occur in Africa [70], meaning the current findings are particularly important regarding the assessment and treatment of CALHIV population in African countries. Furthermore, the remaining five studies act to represent a global perspective, with reviews from Asia, Europe, North America and South America. With the majority of these studies having good quality, the present review boasts sufficient geographical representation.

### Recommendations

The primary focus of the current systematic review was on the challenges faced by CALHIV. While the identification of such challenges is important in its own right, it is essential that protective factors associated with stigma are also addressed. Previous research has suggested that perceived social support, future orientation, trusting relationships and self-efficacy may all help mitigate the negative effects of HIV-related stigma among HIV-affected populations and populations living with HIV [71, 72]. This is an important factor to consider in the interpretation of stigma-related challenges faced by CALHIV. It is recommended that future research considers the effect of such possible protective factors and a review incorporating these two domains may be warranted.

A significant proportion of HIV-stigma interventions are targeted towards public attitudes and reducing enacted stigma, rather than targeting the individuals living with HIV [22]. This is a practice that must be continued, due to much of the findings relating to peers and their influence on CALHIV. Due to peer-group related pressures being such a central aspect within many of the themes, it is essential that public attitude interventions continue to be available, particularly in schools and clubs where many children congregate together. However, the lack of individually-targeted interventions is a major issue as four out of five of the themes focused on the effects of anticipated and internalised stigma, with only social exclusion incorporating the effects of enacted stigma. Furthermore, enacted stigma was only recounted by children and adolescents in six of the studies, as opposed to anticipated and internalised stigma featuring in eleven and ten of the studies, respectively. These challenges associated with anticipated and internalised stigma warrant more individualised intervention approaches [58]. Moreover, interventions need to target the immediate families of CALHIV in order to address the development of negative core beliefs [22, 63]. It is simply not enough to improve public attitudes without addressing personal beliefs. In line with this view, it is a recommendation of this review that in the development of interventions specifically towards CALHIV, special attention should be given to the age-specific challenges of feelings of abnormality and disclosure-related anxiety.

Furthermore, as medication adherence may also be a larger issue in CALHIV than adults living with HIV, due to anticipated stigma, individual interventions paired with public interventions may help decrease nonadherence by addressing both the public attitudes surrounding HIV and the internalisation of such attitudes by the CALHIV. Sweeney and Vanable [73] conducted a review which linked stigma to difficulties in medication adherence in people living with HIV, due to mental health difficulties, reduced self-efficacy and disclosure concerns. As such, cognitive-behavioural interventions for the affected individuals were recommended to target internalised beliefs and promote skill development when faced with external stigma. However, future research regarding these suggested interventions is recommended, due to the paucity of theoretically sound interventions present in HIV stigma interventions for those living with HIV [74].

Such a mixture of interventions may help CALHIV to reap internal rewards, but also help dismantle the societal constructs that enforce this stigma in wider society. While the addition of individual interventions may help CALHIV cope with stigma, the onus must not be solely placed upon CALHIV, and the continuation of public interventions must continue. Particularly, the role of society can be emphasised in individual interventions to ensure the individual does not place further blame upon themselves. More generally, these interventions should be delivered with caution, ensuring that it is not left to the individual to solve their own problem, in order to reap the benefits. Furthermore, though beyond the realms of the current review, it is recommended that the interactions between enacted stigma and anticipated/internalised stigma is explored. Identified interaction may greatly inform the provision of public and individual interventions and the extent to which enacted stigma may exacerbate feelings of anticipated/internalised stigma.

## Conclusion

To the research team’s knowledge, this is the first systematic review to address the health and wellbeing challenges associated with HIV-related stigma from the perspectives of CALHIV. The findings suggest that children and adolescents face challenges surrounding disclosure-related anxiety, medication adherence, feelings of abnormality, metal health issues and social exclusion. As such, HIV-related stigma appears to be hazardous to the health and wellbeing of CALHIV. In particular, anticipated and internalised stigma appear to be the main drivers of all the aforementioned challenges, with the exception of social exclusion. These results greatly emphasise a need for interventions targeted towards these specific aspects of stigma and its associated challenges. Further support for such interventions lies within the specific challenges identified facing CALHIV. Disclosure-related anxiety and feelings of abnormality are challenges distinct to the childhood and adolescence experience, possibly due to the additional societal pressure faced by young people to fit in with the crowd. These findings support the idea that CALHIV’s voices are vital in research involving this population, as they can provide valuable insights into their lives that may not be generated through a comprehensive analysis involving all ages. Moving forward, it is recommended that CALHIV are at the forefront of the research, with the aim of developing tailored programmes and interventions specific to the needs of this unique cohort. Children and adolescents may now be able to live full lives with a chronic disease, but it needs to be made sure that they are not living lives full of chronic stigma.

## Electronic Supplementary Material

Below is the link to the electronic supplementary material.

Supplementary Material 1

Supplementary Material 2

Supplementary Material 3

## Data Availability

A systematic search strategy was used to identify articles in Embase, PsycInfo, PubMed and CINAHL Complete containing the selected search terms.
