# Supplementary material for: Examining the Relationship Between HIV-Related Stigma and the Health and Wellbeing of Children and Adolescents Living with HIV: A Systematic Review
Source: AIDS Behav. 2023 Mar 14;27(9):3133–49. doi: 10.1007/s10461-023-04034-y (PMC10386953; doi:10.1007/s10461-023-04034-y)
Supplement: Supplementary file 1 — Supplementary Material 1 [file 10461_2023_4034_MOESM1_ESM.docx]

## **Appendix C.** *Extraction Tables*

| **Title** | Barriers to Antiretroviral Medication Adherence in Young HIV-Infected Children |
| --- | --- |
| **Authors (Year)** | Roberts (2005) |
| **Location** | USA |
| **Study Type** | Qualitative |
| **Research Design** | Interviews |
| **Aim** | Exploring the barriers to adhering to medication from the perspectives of children and their guardians |
| **HIV+ Participants** | **Recruitment:** HIV Clinics  **Total *n:*** 9  **Age Range (Mean):** 7-12 years (9.5)  **Female:Male Ratio:** 3:6  **Perinatally-infected:** Unspecified |
| **Other Groups Involved** | Yes - Guardians (*n* = 14) |
| **Type of Analysis** | Thematic analysis |
| **Scales Used (if applicable)** | N/A |
| **Type of Stigma** | Anticipated and internalised stigma inferred from findings |
| **Summary of Results** | Stigma emerged as its own theme. This related to taking medication in privacy and providing cover up lies of its purpose. Reminder of HIV/AIDS also emerged as a theme, highlighting the alienation children felt due to having a stigmatised disease. |
| **Quality Assessment** | Higher Quality |
| **Conflicting Interests** | Not stated |
| **Ethics** | Not stated |
| **Funding** | Funded by grant from State of California, Universitywide AIDS Research Program to the UCLA CARE Centre |

| **Title** | A Qualitative Exploration of Psychosocial Challenges of Perinatally HIV-Infected Adolescents and Families in Bangkok, Thailand |
| --- | --- |
| **Authors (Year)** | Friedman-Nestadt et al. (2018) |
| **Location** | Thailand |
| **Study Type** | Qualitative |
| **Research Design** | FGD & Interviews |
| **Aim** | Explore the psychosocial issues present among Thai perinatally infected HIV+ adolescents and their families to inform future interventions |
| **HIV+ Participants** | **Recruitment:** HIV Clinics  **Total *n:*** 10  **Age Range (Mean):** 13-16 years (14.4)  **Female:Male Ratio:** 5:5  **Perinatally-infected:** Yes |
| **Other Groups Involved** | Yes - Caregivers (*n* = 8), Key Informants (*n =* 6) |
| **Type of Analysis** | Framework Analysis |
| **Scales Used (if applicable)** | N/A |
| **Type of Stigma** | Anticipated and internalised stigma inferred from findings |
| **Summary of Results** | Two themes addressed stigma. Adherence issues arose from worry surrounding taking medication in front of peers. Disclosure highlighted secrecy and fear felt among HIV+ youths towards their peers and partners. |
| **Quality Assessment** | Lower Quality |
| **Conflicting Interests** | Authors declared no conflicting interests |
| **Ethics** | Not stated |
| **Funding** | Funding was provided through multiple grants |

| **Title** | Perceptions and Experiences about Self-Disclosure of HIV Status among Adolescents with Perinatal Acquired HIV in Poor-Resourced Communities in South Africa |
| --- | --- |
| **Authors (Year)** | Madiba & Mokgatle (2016) |
| **Location** | South Africa |
| **Study Type** | Qualitative |
| **Research Design** | Interviews |
| **Aim** | To explore how adolescents with perinatally-infected HIV experience living with HIV and their perceptions related to disclosure to friends and sexual partners. |
| **HIV+ Participants** | **Recruitment:** HIV Clinics  **Total *n:*** 37  **Age Range (Mean):** 12-18 years (15.5)  **Female:Male Ratio:** 22:15  **Perinatally-infected:** Yes |
| **Other Groups Involved** | No |
| **Type of Analysis** | Thematic analysis |
| **Scales Used (if applicable)** | N/A |
| **Type of Stigma** | Anticipated and enacted stigma inferred from findings |
| **Summary of Results** | Two themes addressed stigma. Keeping the stigma encompassed the fear of being gossiped about as a reason for secrecy and the instructions from family for secrecy. Experience of isolation resulted from breaking this secrecy. Self-disclosure addressed fears of rejection and isolation from peers and romantic partners. |
| **Quality Assessment** | Higher Quality |
| **Conflicting Interests** | Authors declared no conflicting interests. |
| **Ethics** | Ethical approval granted by University of Limpopo Research and Ethics Committee, Medunsa Campus. |

| **Title** | Growing up With HIV/AIDS: A Study on Adolescents With HIV/AIDS and Their Family Caregivers |
| --- | --- |
| **Authors (Year)** | Lima & Pedro (2008) |
| **Location** | Brazil |
| **Study Type** | Qualitative |
| **Research Design** | Interviews |
| **Aim** | Exploratory study aiming to identify how the adolescent process occurs in perinatally-infected HIV+ adolescents and how the families perceive this process |
| **HIV+ Participants** | **Recruitment:** HIV Clinics  **Total *n:*** 4  **Age Range (Mean):** 11-14 years (not stated)  **Female:Male Ratio:** 4 females  **Perinatally-infected:** Yes |
| **Other Groups Involved** | Yes - Caregivers (*n* = 3) |
| **Type of Analysis** | Content Analysis |
| **Scales Used (if applicable)** | N/A |
| **Type of Stigma** | Internalised stigma inferred by findings |
| **Summary of Results** | The subcategories of the diagnosis revelation, the adolescent feelings and reactions highlighted non-acceptance of such a stigmatised disease in children and adolescents, paired with negative emotions and feelings of inequality to their peers. Even when moved into the acceptance phase, anguish was still experienced. |
| **Quality Assessment** | Lower Quality |
| **Conflicting Interests** | Not stated |
| **Ethics** | Ethical approval granted by the Research Ethics Committee at the Maternal Child Hospital Presidente Vargas. |
| **Funding** | Not stated |

| **Title** | Experiences of Children Living With HIV and AIDS Following Their Diagnosis Disclosure in Mzuzu, Malawi |
| --- | --- |
| **Authors (Year)** | Phuma-Ngaiyaye & Dartey (2015) |
| **Location** | Malawi |
| **Study Type** | Qualitative |
| **Research Design** | Interviews |
| **Aim** | To explore the experiences of HIV+ children and adolescents following diagnosis disclosure |
| **HIV+ Participants** | **Recruitment:** HIV Clinics  **Total *n:*** 10  **Age Range (Mean):** 10-14 years (not stated)  **Female:Male Ratio:** 5:5  **Perinatally-infected:** Unspecified |
| **Other Groups Involved** | Yes - Caregivers (*n* = 7) |
| **Type of Analysis** | Content analysis |
| **Scales Used (if applicable)** | N/A |
| **Type of Stigma** | Anticipated and enacted stigma inferred by findings |
| **Summary of Results** | One theme, the fear of being stigmatised, highlighted fear of stigmatising by their peers, leading to non-disclosure. Also explored experienced stigma within the family environment. |
| **Quality Assessment** | Higher Quality |
| **Conflicting Interests** | Authors declared no conflicting interests |
| **Ethics** | Ethical approval granted by the National Health Sciences Research Committee in Malawi |
| **Funding** | Funded by Mzuzu University, Malawi |

| **Title** | 'Children Will Always Be Children': Exploring Perceptions and Experiences of HIV-Positive Children Who May Not Take Their Treatment and Why They May Not Tell |
| --- | --- |
| **Authors (Year)** | Kawuma et al. (2014) |
| **Location** | Uganda |
| **Study Type** | Qualitative |
| **Research Design** | Interviews |
| **Aim** | To describe the experience of HIV+ adolescents on antiretroviral therapy in different care environments |
| **HIV+ Participants** | **Recruitment:** HIV Clinics  **Total *n:*** 26  **Age Range (Mean):** 11-13 years (not stated)  **Female:Male Ratio:** 12:14  **Perinatally-infected:** Unspecified |
| **Other Groups Involved** | Yes - Caregivers (*n* = 10), Health Workers (*n* = 5) |
| **Type of Analysis** | Content and Thematic Analysis |
| **Scales Used (if applicable)** | N/A |
| **Type of Stigma** | Anticipated and internalised stigma inferred from findings |
| **Summary of Results** | Fear of being seen by others was identified as a theme. This stemmed from family members encouraging secrecy. This occurred due to a desire to avoid disclosure and resulting stigma. This led to missed doses due to lack of privacy and lack of reminders. |
| **Quality Assessment** | Higher Quality |
| **Conflicting Interests** | Not stated |
| **Ethics** | Not stated |
| **Funding** | Funded by the Department for International Development |

| **Title** | ‘Everyone Has a Secret They Keep Close to Their Hearts’: Challenges Faced by Adolescents Living With HIV Infection at the Kenyan Coast |
| --- | --- |
| **Authors (Year)** | Abubakar et al. (2016) |
| **Location** | Kenya |
| **Study Type** | Qualitative |
| **Research Design** | Interviews |
| **Aim** | To investigate HIV+ adolescents experiences and challenges living with HIV, particularly focusing on psychosocial issues |
| **HIV+ Participants** | **Recruitment:** HIV Clinics  **Total *n:*** 12  **Age Range (Mean):** 12-17 years (14.5)  **Female:Male Ratio:** 3:9  **Perinatally-infected:** Yes |
| **Other Groups Involved** | Yes - Caregivers (*n* = 11), Health Workers (*n* = 8), Teachers (*n* = 6), Non-infected Adolescents (*n* = 7) |
| **Type of Analysis** | Framework Analysis |
| **Scales Used (if applicable)** | N/A |
| **Type of Stigma** | Anticipated stigma inferred from findings |
| **Summary of Results** | The theme of poor handling of disclosure discussed how partial disclosure appeared to be the preferred method of disclosure, however this led to unfavourable relationship consequences and evoked consistent anxiety. Many feared full disclosure would cause rejection and isolation. Problems managing medicine existed as a result of this. |
| **Quality Assessment** | Higher Quality |
| **Conflicting Interests** | Authors declared no conflicting interests |
| **Ethics** | Ethical approval granted by the Kenya Medical Research Institute National Scientific and Ethical Committee |
| **Funding** | Funded by multiple sources |

| **Title** | Hear Our Voices: A Photovoice Project With Children Who Are Orphaned and Living With HIV in a Ugandan Group Home |
| --- | --- |
| **Authors (Year)** | Fournier et al. (2014) |
| **Location** | Uganda |
| **Study Type** | Qualitative |
| **Research Design** | Photovoice & FGD |
| **Aim** | Explore the experiences of HIV+ orphaned children living in a group home |
| **HIV+ Participants** | **Recruitment:** HIV/AIDS Children’s Group Home  **Total *n:*** 13  **Age Range (Mean):** 12-18 years (not stated)  **Female:Male Ratio:** 5:8  **Perinatally-infected:** Probable |
| **Other Groups Involved** | No |
| **Type of Analysis** | SHOWeD Framework and Matrix Methodology techniques described by Miles and Huberman (1994) |
| **Scales Used (if applicable)** | N/A |
| **Type of Stigma** | Enacted and internalised stigma inferred from findings |
| **Summary of Results** | Subtheme of stigma and discrimination highlighted the negative experiences of those in the children’s home being signalled out as having AIDS. In some cases suicidality was alluded to as a result of this enacted stigma. Emotional challenges such as anger, bullying, abuse, accusation of criminal activity were also addressed. |
| **Quality Assessment** | Lower Quality |
| **Conflicting Interests** | Not stated |
| **Ethics** | Ethical approval was granted by Canada: Mount Royal University, the University of Alberta, Makerere University and the Uganda National Council for Science and Technology |
| **Funding** | Not stated |

| **Title** | HIV Stigma: Perspectives from Kenyan Child Caregivers and Adolescents Living With HIV |
| --- | --- |
| **Authors (Year)** | McHenry et al. (2017) |
| **Location** | Kenya |
| **Study Type** | Qualitative |
| **Research Design** | FGD |
| **Aim** | To characterise the understanding, experience and impact of HIV-related stigma on adolescents living with HIV and exploring how they think this stigma may be measured and reduced |
| **HIV+ Participants** | **Recruitment:** HIV Clinics  **Total *n:*** 40  **Age Range (Mean):** 10-15 years (13)  **Female:Male Ratio:** 28:12  **Perinatally-infected:** Unspecified |
| **Other Groups Involved** | Yes - Caregivers (*n* = 53) |
| **Type of Analysis** | Constant Comparative Analysis |
| **Scales Used (if applicable)** | N/A |
| **Type of Stigma** | Anticipated, enacted and internalised all explicitly stated |
| **Summary of Results** | Prominent role of HIV/AIDS stigma, Impact of HIV/AIDS stigma on treatment and prevention and perspectives on identifying, measuring and reducing HIV/AIDS stigma were all relevant themes. Anticipated stigma most frequently reported, particularly regarding the loss of relationships due to their HIV status. This also affected medication adherence due to secrecy and physical health as a result of this. Internalised stigma included participants reporting low self-esteem, being unworthy and hating themselves. Fewer participants reported experiencing stigma. |
| **Quality Assessment** | Higher Quality |
| **Conflicting Interests** | Authors declared no conflicting interests |
| **Ethics** | Ethical approval granted by Indiana University School of Medicine and Moi University School of Medicine and MTRH in Eldoret, Kenya |
| **Funding** | Grant entitled “Stigma in AIDS Family Inventory” from the National Institute for Mental Health, Bethesda, Maryland. |

| **Title** | Growing Up: Perspectives of Children, Families and Service Providers Regarding the Needs of Older Children With Perinatally-Acquired HIV |
| --- | --- |
| **Authors (Year)** | Fielden et al. (2006) |
| **Location** | Canada |
| **Study Type** | Qualitative |
| **Research Design** | FGD & Interviews |
| **Aim** | To examine the specific needs of older children living with perinatally acquired HIV |
| **HIV+ Participants** | **Recruitment:** HIV Clinics  **Total *n:*** 10  **Age Range (Mean):** 9-16 years (not stated)  **Female:Male Ratio:** 5:5  **Perinatally-infected:** Yes |
| **Other Groups Involved** | Yes - Service Providers (*n* = 11), Caregivers (*n* = 11) |
| **Type of Analysis** | Multi-step process guided by principles of qualitative inquiry (Pope & Mays, 1999) |
| **Scales Used (if applicable)** | N/A |
| **Type of Stigma** | Anticipated and enacted stigma explicitly stated, internalised stigma inferred from findings |
| **Summary of Results** | Social stigma and mental health were two key themes incorporating stigma. Social stigma was frequently associated with fear, secrecy, trust, disclosure and isolation. Some participants reported losing friends and being run out of town. Mental health highlighted the atypical worries possessed by HIV+ youth, such as partner rejection. |
| **Quality Assessment** | Higher Quality |
| **Conflicting Interests** | Not stated |
| **Ethics** | Ethical approval granted by university and hospital boards |
| **Funding** | Multiple sources of funding |

| **Title** | Internalized Hiv Stigma, Bullying, Major Depressive Disorder, and High-Risk Suicidality Among HIV-Positive Adolescents in Rural Uganda |
| --- | --- |
| **Authors (Year)** | Ashaba et al. (2018) |
| **Location** | Uganda |
| **Study Type** | Quantitative |
| **Research Design** | Questionnaire & Interviewed Scales |
| **Aim** | To estimate the associations between internalised stigma, bullying, major depressive disorder and suicidality in HIV+ adolescents |
| **HIV+ Participants** | **Recruitment:** HIV Clinics  **Total *n:*** 224  **Age Range (Mean[Standard Deviation]):** 13-17 years (14.8[1.4])  **Female:Male Ratio:** 131:93  **Perinatally-infected:** Probable |
| **Other Groups Involved** | No |
| **Type of Analysis** | Bivariate analysis and multiple regression |
| **Scales Used (if applicable)** | Mini International Neuropsychiatric Interview for Children and Adolescents (MINI-KID; Sheehan et al., 2010)  Internalised AIDS Related Stigma Scale (Kalichman et al., 2009)  Social and Health Assessment Peer Victimization Scale (Ruchkin et al., 2004) |
| **Type of Stigma** | Internalised stigma measured by scale |
| **Summary of Results** | Internalized stigma was associated with major depressive disorder and suicidality. Estimated associations remained statistically significant after multivariable adjustment only in any suicidality.  **Bivariate Analysis:** major depressive disorder associated with internalized stigma (OR 1.31; 95% CI 1.01–1.73; p = 0.04)  Any suicidality also associated with internalized stigma (OR 1.33; 95% CI 1.07–1.66; p = 0.009).  **Multiple Regression:** Major depressive disorder MRM did not have a statistically significant association with internalised stigma (AOR 1.27; 95% CI 0.94–1.73; p = 0.12)  Any Suicidality MRM stigma did have a statistically significant association with stigma (AOR 1.30; 95% CI 1.03–1.30; p = 0.02).  In sensitivity analysis, high risk suicidality the DV in the regression model, stigma did not have a significant association (AOR 1.35; 95% CI 0.91–2.01; p = 0.13). |
| **Quality Assessment** | Good |
| **Conflicting Interests** | Authors declared no conflicting interests |
| **Ethics** | Comply with the ethical standards of the national and institutional committees on human experimentation and with the Helsinki Declaration of 1975 |
| **Funding** | Multiple funders |

| **Title** | HIV-Related Stigma and Health-Related Quality of Life Among Children Living With HIV in Sweden |
| --- | --- |
| **Authors (Year)** | Rydström et al. (2016) |
| **Location** | Sweden |
| **Study Type** | Quantitative |
| **Research Design** | Questionnaire |
| **Aim** | To describe the experiences of HIV-related stigma and Health Related Quality of Life in children living with perinatally acquired HIV and to investigate the relationship between these two domains |
| **HIV+ Participants** | **Recruitment:** HIV Clinics  **Total *n:*** 58  **Age Range (Mean[Standard Deviation]):** 8-18 years (13.9[1.5])  **Female:Male Ratio:** 27:31  **Perinatally-infected:** Yes |
| **Other Groups Involved** | No |
| **Type of Analysis** | Structural Equation Modelling |
| **Scales Used (if applicable)** | HIV Stigma Scale for Children (HSSC-8; Wiklander et al., 2013)  HRQoL - DISABKIDS Chronic Generic Module (DCGM-37; Bullinger et al., 2002)  Disclosure - three specific study questions developed concerning disclosure of HIV. Covered if told, who told. |
| **Type of Stigma** | Anticipated and internalised measured by scale |
| **Summary of Results** | Disclosure concerns were the most prominent stigma related association.  Stigma and HRQoL strongly negatively correlated (standardized β = –0.790; p = 0.017). The interpretation of this standardized coefficient is that if HIV stigma increases by one SD, HRQoL will decrease by 0.79 SD. Age, sex or treatment did not show any statistically significant contribution to the model. |
| **Quality Assessment** | Good |
| **Conflicting Interests** | Authors declared no conflicting interests |
| **Ethics** | Ethical approval granted by the Regional Ethical Review Board of Stockholm |
| **Funding** | This study was co-funded by the Doctoral School of Health Care Sciences, Karolinska Institutet, the Strategic Research Program in Care Sciences, Karolinska Institutet and the Kempe-Carlgrenska Foundation. |

| **Title** | Predictors of Mental Health Problems in Adolescents Living With HIV in Namibia |
| --- | --- |
| **Authors (Year)** | Gentz et al. (2017) |
| **Location** | Namibia |
| **Study Type** | Quantitative |
| **Research Design** | Questionnaire |
| **Aim** | To examine the psychological impact of HIV on infected adolescents, particularly focusing on mental health |
| **HIV+ Participants** | **Recruitment:** HIV Clinics  **Total *n:*** 99  **Age Range (Mean[Standard Deviation]):** 12-18 years (14.33[1.8])  **Female:Male Ratio:** 52:47  **Perinatally-infected:** Probable |
| **Other Groups Involved** | No |
| **Type of Analysis** | Pearson’s or Spearman’s correlation for continuous scores. Hierarchical multiple linear regression. |
| **Scales Used (if applicable)** | SDQ Youth Version (Goodman, 1997)  Shortened Version of Berger's HIV Stigma Scale (Wright et al., 2007). |
| **Type of Stigma** | Anticipated, enacted and internalised all measured by scale |
| **Summary of Results** | **Correlations:** There was a positive correlation between total  stigma reported and total difficulties (.356, p <.01), emotional symptoms (.36, p <.001), peer problems (.226, p <.05) and conduct problems (.235, p <.05), with internalised stigma having a positive correlation with total difficulties (.379, p < .001), emotional symptoms (.399, p < .001) and conduct problems (.236, p < .05). Personalised stigma was only associated with peer problems (.294, p < .01). Publical attitude was associated with total difficulties (.285, p < .01), emotional smptoms (.304, p < .01), and conduct problems (.216, p < .05).  **Multiple Regression:** Child assets (t = 2.25, p = .027, b = .223) and stigma (t = 2.62, p = .011, b = .261) significantly predicted total difficulties scores.  Stigma was the only significant predictor in predicting emotional symptoms (t = 3.14, p = .002, b = .308). |
| **Quality Assessment** | Good |
| **Conflicting Interests** | Authors declared no conflicting interests |
| **Ethics** | Ethical approval granted by the Institutional Review Board of the Universidad Complutense de Madrid and the Ministry of Health and Social Services in Namibia. |
| **Funding** | Funded through AECID Scholarship |

| **Title** | High Self-Reported Non-Adherence to Antiretroviral Therapy Amongst Adolescents Living With HIV in Malawi: Barriers and Associated Factors |
| --- | --- |
| **Authors (Year)** | Kim et al. (2017) |
| **Location** | Malawi |
| **Study Type** | Quantitative |
| **Research Design** | Questionnaire |
| **Aim** | To investigate self-reported adherence and barriers to adherence in HIV+ adolescents and to examine the factors affecting suboptimal adherence |
| **HIV+ Participants** | **Recruitment:** HIV Clinics  **Total *n:*** 519  **Age Range (Mean[Standard Deviation]):** 12-18 years (14.5[2])  **Female:Male Ratio:** 290:229  **Perinatally-infected:** Yes |
| **Other Groups Involved** | No |
| **Type of Analysis** | Fisher’s Test & Multivariate Analysis |
| **Scales Used (if applicable)** | Adherence self-report measure (Chesney et al., 2000; MacDonnell et al., 2016)  19-item checklist of barriers to adherence (Buchanan et al., 2012) |
| **Type of Stigma** | Anticipated stigma measured by scale |
| **Summary of Results** | 9.8% of individuals who reported non-adherence reported stigma outside the home as a barrier. 5.2% of them reported stigma inside the home as a barrier. Non-adherent individuals reported stigma outside the home as a barrier significantly more than adherent individuals (*p* < .001). |
| **Quality Assessment** | Acceptable |
| **Conflicting Interests** | Not explicitly stated |
| **Ethics** | Ethical approval granted by the National Health Sciences Research Committee (NHSRC) of Ministry of Health in Malawi and the Baylor College of Medicine Institutional Review Board in the USA |
| **Funding** | Multiple sources of funding |

| **Title** | Mental health and Functional Competence in the Cape Town Adolescent Antiretroviral Cohort |
| --- | --- |
| **Authors (Year)** | Hoare et al. (2019) |
| **Location** | South Africa |
| **Study Type** | Quantitative |
| **Research Design** | Questionnaire |
| **Aim** | To describe the mental health functioning in perinatally infected HIV+ adolescents |
| **HIV+ Participants** | **Recruitment:** HIV Clinics  **Total *n:*** 204  **Age Range (Mean[Standard Deviation]):** 9-11 years (10.38[.88])  **Female:Male Ratio:** 104:100  **Perinatally-infected:** Yes |
| **Other Groups Involved** | Y - non-infected control (*n* = 44) |
| **Type of Analysis** | Mann-Whitney U, Linear Regression Models |
| **Scales Used (if applicable)** | Beck Youth Inventories  Child Behaviour Checklist (Ivanova et al., 2007)  The Life Events Questionnaire  HIV-related stigma questions rated between 1 = Not at all and 3 = All the time. |
| **Type of Stigma** | General: HIV-related Stigma |
| **Summary of Results** | More HIV-related stigma was associated with depression, anger, and disruptive behavior.  **Unadjusted B (95% CI) P Adjusted B (95% CI) P**  **Depression:** 9.72 (2.21 to 17.24) 0.012 9.93 (2.88 to 16.98) 0.006  **Anger:** 7.89 (20.27 to 16.06) 0.058 8.18 (0.24 to 16.13) 0.044  **Disruptive:**  10.67 (3.77 to 17.57) 0.003 11.13 (4.27 to 17.99) 0.002 |
| **Quality Assessment** | Good |
| **Conflicting Interests** | Authors declared no conflicting interests |
| **Ethics** | Ethical approval granted by the University of Cape Town’s Faculty of Health Sciences research ethics committee |
| **Funding** | Not stated |
