# Supplementary material for: Examining the Relationship Between HIV-Related Stigma and the Health and Wellbeing of Children and Adolescents Living with HIV: A Systematic Review
Source: AIDS Behav. 2023 Mar 14;27(9):3133–49. doi: 10.1007/s10461-023-04034-y (PMC10386953; doi:10.1007/s10461-023-04034-y)

## **Appendix A.** *Example of PsycInfo Search Strategy*

The search strategy for PsycInfo was as follows: (ylwha OR childhood HIV OR HIV-infected children OR perinatal HIV OR perinatally HIV-infected children OR adolescent HIV OR youth living with HIV OR children living with HIV or adolescents living with HIV) AND (stigma OR stigmatisation OR stigmatization OR prejudice OR discrimination OR HIV/AIDS-related stigma OR HIV-related stigma OR AIDS-related stigma OR perceived stigma OR experiences stigma OR anticipated stigma OR self-stigma OR shame OR stereotyp*).

**Figure 2.**

*Screen Capture of the Search Completed on Psycinfo*


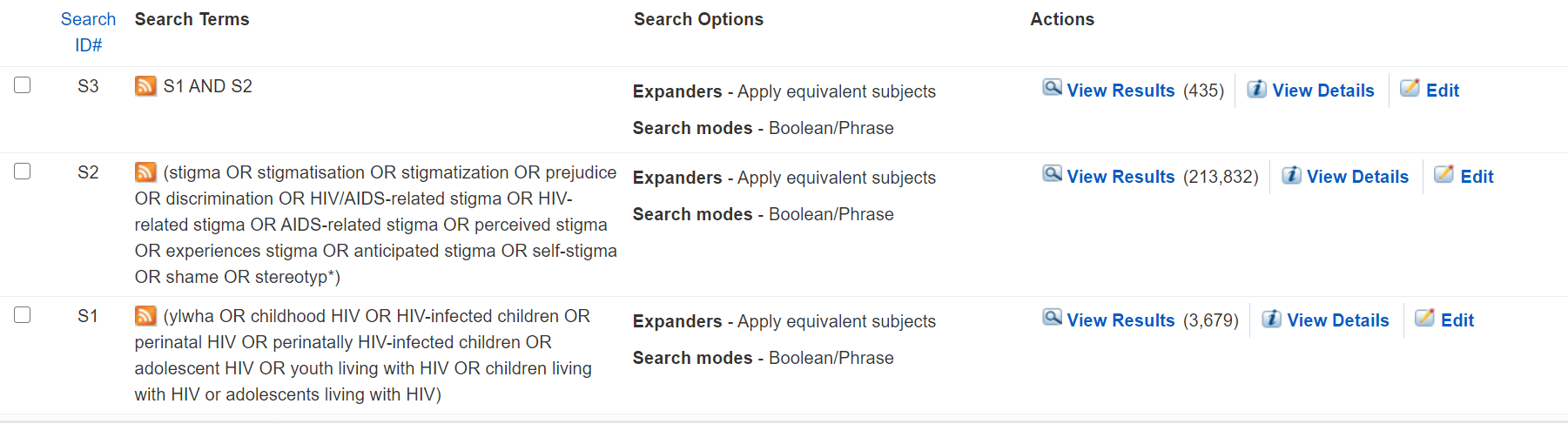

Supplement: Supplementary file 2 — Supplementary Material 2 [file 10461_2023_4034_MOESM2_ESM.docx]
