# Supplementary material for: Examining the Relationship Between HIV-Related Stigma and the Health and Wellbeing of Children and Adolescents Living with HIV: A Systematic Review
Source: AIDS Behav. 2023 Mar 14;27(9):3133–49. doi: 10.1007/s10461-023-04034-y (PMC10386953; doi:10.1007/s10461-023-04034-y)
Supplement: Supplementary file 3 — Supplementary Material 3 [file 10461_2023_4034_MOESM3_ESM.docx]

## **Appendix B.** *Line-by-Line Coding Example*

Line-by-line coding was conducted by hand on the results, key quotes and discussion sections of the qualitative studies. Coding was also applied to the variables used in quantitative analysis to aid synthesis of the two methods. A sample of the coding of four studies is provided below which reflects elements of each of the finalised themes. The hand coding was adapted and translated over to a computerised version for clarity purposes.

**Key:** *anticipated stigma, **enacted stigma, ***internalised stigma

**Disclosure-related Anxiety**

**Medication Adherence**

**Feelings of Abnormality**

**Mental Health Issues**

**Social Isolation**

**Kawuma et al. (2014)**

'Children Will Always Be Children': Exploring Perceptions and Experiences of HIV-Positive Children Who May Not Take Their Treatment and Why They May Not Tell

| **Extract:**  “Even before knowing status children were encouraged to take drugs in private, out of view of other children and non-family members. This continued into them knowing their status. This behaviour was largely shaped by a desire to avoid deductive disclosure and the resulting stigma. Children and their carers wanted to keep their status and drug-taking a secret. Children who found it hard to find a private place would miss their dose because they were afraid of people interrupting and finding out their status. Keeping this secret was an obstacle to drug adherence, not only due to fear of someone seeing, but also no one to remind the child to take their dose. Being reminded to take a dose disrupts children's desire to be seen as normal children.”  “However, we found that non-adherence was more commonly a consequence of children strategically trying to maintain their relationships. For example, children may cite playing as a reason for poor adherence. Play does not necessarily cause them to forget to take their drugs, but rather they do not want their friends to know that they need to take treatment lest this be a cause of deductive disclosure of their status.” | *Association between medication and secrecy  *Secrecy influenced by caregiver  *Medication acting as HIV signifier  Forgetting dose due to secrecy  *Secrecy  *Medication acting as HIV signifier |
| --- | --- |
| **Key Quotes:**  “Children [from the neighbourhood] would come [to my] home so early in the morning and when the time for taking drugs comes I would take it while they are there and when they see me taking it they ask me and when I tell them they start teasing me. So when they come I don’t find time to take it yet even the tins [with the drugs] are in the sitting room” (11 year-old male).  “On that day, mother wasn’t at home and my brother was in senior one and would come home late…I also realised that I had forgotten.” (12 year-old male) | *****Bullying from peers  *Adherence issues mediated by medication acting as HIV signifier  Forgotten dose due to secrecy |

**McHenry et al. (2017)**

HIV Stigma: Perspectives from Kenyan Child Caregivers and Adolescents Living With HIV

| **Extract:**  “In most discussions about H/A stigma, stigma described would be perceived stigma. Adolescents had similar fears about discrimination and social isolation, with the most significant being that of losing friends, diminished social interactions, and loss of respect among peers. Most of the fears about perceived stigma focused on the loss of social interactions, but participants also described fears of losing resources because of stigma. Fewer participants described instances of lived experiences of H/A stigma (or ‘‘enacted’’ stigma)”  “A common manifestation of internalized stigma was having low selfesteem, and participants described feelings of ‘‘hating themselves’’ and ‘‘insult[ing] themselves in their hearts.’’ Participants described sometimes feeling unworthy of the very social interactions with family and friends that they so feared losing due to their HIV status.”  “Both adolescents and caregivers described not telling others they are on a medication, hiding medicines at their homes, and taking the medication in secret—all of which sometimes led to nonadherence.”  “Stigma could create psychological stress, which then results in physical illness or ill appearance, largely because of nonadherence to HIV treatment. Participants identified adherence as being closely associated with H/A stigma, high levels of stigma = less likely to be adherent, with subsequent physical illness or weight loss altering the physical appearance. Finally, participants thought that psychological distress in the form of feeling depressed, ‘‘stressed,’’ ‘‘restless,’’ or ‘‘losing hope’’ were all associated with H/A stigma.” | *Fear surrounding isolation and loss of relationships due to disclosure  **Discrimination from others less prevalent  ***Heightened negative emotions towards self  ***Self-hatred  ***Unworthiness, unequal to peers  *Secrecy  *Medication acts as HIV signifier  *Physical ill health repercussions  *Physical ill health repercussions  ***Low mood, hopelessness, stress |
| --- | --- |
| **Key Quotes:**  ‘‘(They) will hate you and will be chasing you away.” (Adolescent) | *Fear of disclosure repercussions on relationships |

**Fielden et al. (2008)**

Growing Up: Perspectives of Children, Families and Service Providers Regarding the Needs of Older Children With Perinatally-Acquired HIV

| **Extract:**  “Social stigma was emphasized as a negative and destructive influence on the well-being of the HIVinfected children and families. It was associated with concepts of fear, secrecy, trust, disclosure and isolation. Two of the oldest children said that having to keep their status a secret was a bad part of their lives. Context-specific, in home towns children are reluctant to disclose because they anticipated and/or had experienced negative consequences such as losing friends or being run out of town.”  “Despite various supports, the children have worries and anxieties that most other children their age do not have, such as being rejected by a girlfriend or boyfriend when they disclose their HIV status.”  “Stigma is a dominant theme. The children and youth with perinatally-acquired HIV targeted in this study are best characterized as a population ‘in hiding’ due the experience and fears of HIV-associated stigma.” | *****Disclosure associated with relational loss  *Negative effects of secrecy  *General disclosure-related anxiety  **Peer and community isolation as a result of status  Abnormality, unequal experience to peers  *Fear of partner rejection  *Secrecy of status |
| --- | --- |
| **Key Quotes:**  “A lot of people know who I am but I don’t really want to be like one of those people who are the centre of attention and everybody is like, ‘‘Oh, look! There’s the girl with HIV.’’’ and ‘I’d rather people see me as Lucy, than people see: ‘‘Oh, Lucy with HIV.’’’ (Older female)  “It’s nice to go to camp (for HIV-affected children) because like I don’t tell any of my friends here that I have HIV. They don’t know anything of that, right. I’m just a regular kid” (Child) | ***Desire for normalcy  ***Feelings of inequality in everyday life |

**Madiba & Mokgatle (2016)**

Perceptions and Experiences about Self-Disclosure of HIV Status among Adolescents with Perinatal Acquired HIV in Poor-Resourced Communities in South Africa

| **Extract:**  “Fear of being gossiped about and stigmatized was an important consideration for keeping their HIV status secret. Some adolescents are given instructions from family to keep secret. Disclosure to friends resulted in stigmatization for some - the experience of this led to secrecy and emotional trauma.”  “Adolescents expressed fear that disclosure to romantic partners and friends would lead to rejection and isolation. Their comments suggested that they feared that their romantic partners and friends would react in a negative way.”  “Maintaining secrecy was also a way to protect themselves from stigma and isolation from others within the home and school environment.”  “Desire to control outward self-disclosure of their HIV status due to negative perceptions of disclosure. This informs disclosure guidelines in interventions. As most adolescents desired to live a normal healthy life,they perceived onward self-disclosure negatively and felt that they would be treated differently if they disclosed their HIV status to friends and romantic partners. Consequently, most maintained secrecy in order to be accepted by their peers. In their context, peer acceptance was much more important than disclosure of their HIV status, which most felt had nothing to do with their friends.” | *Fear of community reaction to status  **Negative repercussions from peers due to status  **Mental ill health  *Fear of peer isolation  *Fear of partner rejection  *Secrecy as a protective factor  *General disclosure-related anxiety  *,***Desire for normalcy, feelings of inequality  *Need to fit in  ***Feelings of inequality |
| --- | --- |
| **Key Quotes:**  “I did not know whom to tell, my mother was always crying, always under stress, and I did not know whom to tell. I told myself that I have to break the silence once and for all, then I told my friends, and they started isolating me.” (14 year-old female)  “I told my friend, and after I told him, he told others; whenever I pass them they bothered me.” (15 year-old male)  “If I tell him, he will leave me.” (18 year-old female) | **Peer isolation  **Bullying related to HIV status  *Fear of partner rejection |
